# Ultrasensitive detection of carbendazim pesticide in tea leaves using a green Ag/CuO(Cu_2_O) nanocomposite-based SERS sensor: role of metal/semiconductor transition in sensing performance†

**DOI:** 10.1039/d5ra00846h

**Published:** 2025-05-27

**Authors:** Dong Thi Linh, Quan-Doan Mai, Dang Thi Hanh Trang, Nguyen Tuan Anh, Xuan Hoa Vu, Anh-Tuan Le

**Affiliations:** a Phenikaa University Nano Institute (PHENA), Phenikaa University Hanoi 12116 Vietnam doan.maiquan@phenikaa-uni.edu.vn tuan.leanh@phenikaa-uni.edu.vn; b Faculty of Fundamental Sciences, Thai Nguyen University of Technology 666 3/2 Road Thai Nguyen City 24000 Vietnam; c Institute of Science and Technology, TNU-University of Sciences Tan Thinh Ward Thai Nguyen City 24000 Vietnam; d Faculty of Materials Science and Engineering (MSE), Phenikaa University Hanoi 12116 Vietnam

## Abstract

Surface-enhanced Raman spectroscopy (SERS) is increasingly recognized as a powerful tool for analytical applications, especially in food safety, due to its ability to detect molecular fingerprints even at the single-molecule level. Developing SERS substrates that offer not only high sensitivity but also reliability and practicability is critical for transitioning SERS from a laboratory-based technique to practical field applications. In this study, we present an outstandingly sensitive, reliable, and practical Ag/CuO nanocomposite SERS substrate, fabricated through a simple green electrochemical method. The Ag/CuO substrate demonstrates remarkable sensitivity, detecting carbendazim (CBZ), a hazardous pesticide widely used in tea leaves, at an ultra-low limit of 8.85 × 10^−11^ M, outperforming bare Ag substrate, which only reaches 10^−6^ M. The high reliability of the Ag/CuO substrate is confirmed by excellent repeatability and reproducibility, with a relative standard deviation (RSD) of less than 10%. Practicability was validated through the direct detection of CBZ in fresh tea leaves, yielding sharp recovery values of 85% to 106%. Additionally, the SERS enhancement mechanism was explored by comparing the performance of Ag, Ag/Cu_2_O, and Ag/CuO substrates, revealing the critical role of metal (Ag) and semiconductor (Cu_2_O, CuO) transitions in overall sensing performance. These findings underscore the potential of Ag/CuO nanocomposites for ultrasensitive pesticide detection in real-world agricultural environments and highlight the importance of metal/semiconductor transitions in designing more efficient SERS substrates. This paves the way for the development of versatile, field-ready SERS platforms applicable to a wide range of analytical and environmental monitoring needs.

## Abstract 2

In this study, we developed an ultrasensitive and practical Ag/CuO-based SERS substrate for reliable detection of carbendazim in tea leaves, highlighting the synergistic enhancement from metal/semiconductor transitions.

## Graphical abstract

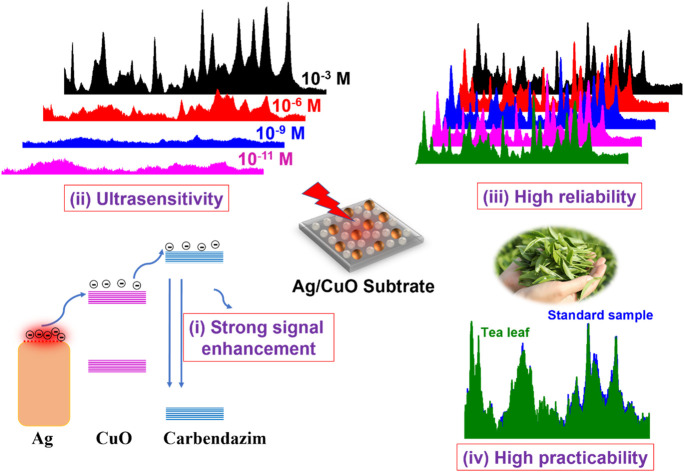

## 1. Introduction

Surface-enhanced Raman spectroscopy (SERS) has emerged as a powerful technique for chemical analysis, particularly in fields like food safety, where accurate, ultra-sensitive detection of harmful chemical residues is critical.^1–3^ The ability of SERS to provide molecular fingerprint information through characteristic Raman vibrational spectra, even at single-molecule levels,^4^ makes it especially useful for detecting trace amounts of pesticides in food,^5,6^ which can pose significant health risks despite their low concentrations. However, accurately detecting these substances remains challenging due to the complex nature of food matrices, which often introduce interference. The high sensitivity of SERS is driven by the interaction between light, nanostructures, and target molecules, with nanostructures playing a central role in enhancing the Raman scattering effect.^3,7,8^ Two well-established mechanisms – electromagnetic (EM) and chemical (CM) – govern this enhancement.^2,9,10^ In response, recent research has focused on optimizing SERS substrates through the design and synthesis of a wide variety of nanostructures, particularly those based on noble metals such as silver (Ag), gold (Au), and copper (Cu).^2,3,11^ Metal/semiconductor-based SERS substrates, in particular, have demonstrated remarkable sensing performance due to the synergistic combination of EM and CM mechanisms.^12–14^ The metal component, characterized by its surface plasmon resonance (SPR), provides EM enhancement, while the presence of the semiconductor facilitates more efficient charge transfer (CT) among the metal, semiconductor, and analyte due to better energy level alignment enabled by the hybrid metal–semiconductor structure.

Semiconductors like titanium dioxide (TiO_2_), zinc oxide (ZnO), and copper oxide (CuO) have been commonly integrated with noble metals in SERS substrates, producing excellent results across various applications.^12,15–17^ For instance, Huang *et al.* developed Ag/TiO_2_ nanocomposites for biosensing, which exhibited strong Raman signal enhancement due to the synergistic combination of EM enhancement from Ag and CM enhancement (specifically CT) from TiO_2_, thereby achieving high sensing performance with detection limits as low as 1.2 × 10^−5^ U mL^−1^ for uracil DNA glycosylase.^18^ Xue *et al.* designed three-dimensional Ag/TiO_2_ aerogels that achieved a detection limit of 10^−11^ M for 4-mercaptobenzoic acid,^19^ attributed not only to the electromagnetic hot spots generated by the porous 3D structure but also to enhanced CM enhancement through multiple interband CT pathways, enabled by the presence of mesoporous TiO_2_ nanoparticles, which improved CT efficiency between the Ag, TiO_2_, and 4-mercaptobenzoic acid, thereby synergistically amplifying the overall SERS response. Yang *et al.* reported core–shell Ag/TiO_2_ substrates that detected thiram pesticide at levels as low as 1.15 × 10^−10^ M, better than those achieved by pure Ag substrates. This improvement resulted from the synergistic effect between the EM enhancement of the Ag core and the CM contribution of the TiO_2_ shell, which facilitated CT and preserved the integrity of S–S bonds in thiram, ensuring both sensitivity and stability.^20^ Following the same design concept based on the synergistic EM and CM enhancement mechanisms, Pal and *et al.* fabricated Ag/ZnO/Au-based three-dimensional SERS substrates capable of detecting lambda DNA at concentrations as low as 10 ng μL^−1^.^21^ Similarly, Yang *et al.* developed CuO@AgNPs nanozyme screen-printed electrodes with excellent SERS performance, detecting crystal violet at a limit of 3 × 10^−10^ M.^22^ These studies demonstrate not only the effectiveness of metal/semiconductor-based SERS substrates but also the potential for further development, particularly in food safety, where ultra-high sensitivity is essential. Additionally, reliability and practicability must be considered to meet the demands of real-world applications. Selecting the appropriate semiconductor for each metal/semiconductor system is key to optimizing SERS performance, as it offers guidance for the design of substrates tailored to specific research objectives.

In this study, we aim to apply a metal/semiconductor-based SERS substrate – Ag/CuO nanocomposite – in food safety applications, specifically for the detection of carbendazim (CBZ) pesticide residues in fresh tea leaves. Our approach involves a comprehensive evaluation of the sensitivity, reliability, and practicability of this substrate to ensure optimal sensor performance. We designed experiments using three types of SERS substrates: bare Ag nanoparticles (NPs), Ag/Cu_2_O nanocomposite, and Ag/CuO nanocomposite, to investigate the SERS enhancement mechanisms and determine the optimal semiconductor for our system. In terms of sensing performance, the Ag/CuO nanocomposite substrate exhibited the highest sensitivity, achieving an ultra-low detection limit of 8.85 × 10^−11^ M for CBZ, far outperforming the bare Ag substrate, which only reached 10^−6^ M. Furthermore, the Ag/CuO substrate demonstrated high reliability and practicability. It also showed remarkable performance in detecting CBZ in real tea samples across a concentration range of 10^−4^ to 10^−10^ M, with sharp recovery rates between 85% and 106%. Mechanistically, the superior sensitivity of the Ag/CuO nanocomposite substrate for CBZ highlights the critical role of energy alignment in the metal/semiconductor transition and its overall contribution to SERS enhancement. This underscores the importance of semiconductor selection to achieve optimal SERS performance in metal/semiconductor/analyte systems.

## 2. Methods

### 2.1. Materials

Precursors and analytes were purchased from Shanghai Chemical Reagent Co., including oleic acid (C_18_H_34_O_2_, 99%), sodium citrate (Na_3_C_6_H_5_O_7_, 99.9%), ascorbic acid (C_6_H_8_O_6_, 99%), carbendazim (C_9_H_9_N_3_O_2_, 99%), ethanol (C_2_H_5_OH, 98%) and used directly without further purification. Silver (Ag) and copper (Cu) plates (purity: 99.99%) were prepared with dimensions of (100 mm × 5 mm × 0.5 mm). Double distilled water was used for all experiments.

### 2.2. Synthesis and characterizations of Ag/Cu_2_O, Ag/CuO nanocomposites

Ag NPs were synthesized using a simple green electrochemical method detailed in our previous study.^23^ A 200 mL solution of double distilled water containing 6 × 10^−5^ M oleic acid was prepared as the electrolyte. Two silver plates, polished and washed with ethanol, were positioned parallel to each other with a 3 cm gap inside a 200 mL beaker. These rods served as the cathode and anode, connected to a power supply. The electrochemical reaction to form Ag NPs was conducted at room temperature, applying a voltage of 12 V for 60 minutes, with continuous stirring at 200 rpm using a magnetic stirrer. During the reaction, the solution changed from transparent to dark brown, indicating the formation of Ag NPs. The morphology, size, and optical properties of the Ag material were analyzed using field emission scanning electron microscopy (FE-SEM, Hitachi S-4800) operated at an acceleration voltage of 5 kV. The results revealed that the synthesized Ag material were spherical with an average diameter of 16 nm. Further details on the properties of these nanoparticles can be found in our previous study. Likewise, CuO and Cu_2_O NPs were synthesized *via* an electrochemical method using two copper electrodes. Sodium citrate and ascorbic acid served as the electrolytes at varying concentrations to produce the desired CuO and Cu_2_O NPs. Detailed experimental procedures and characterizations are presented in our prior work.^24^ Ag/Cu_2_O and Ag/CuO nanocomposites were fabricated by ultrasonically mixing the synthesized Ag NPs with CuO (or Cu_2_O) NPs for 30 minutes to achieve uniform dispersion. A CuO (or Cu_2_O) content of 10% by total nanocomposite mass was selected to evaluate its SERS enhancement capabilities. Additionally, CuO contents of 5%, 10%, 15%, and 20% were investigated to determine the optimal composition for maximum SERS enhancement.

### 2.3. Substrate preparation, SERS measurements

Aluminum (Al) substrates, with dimensions of 1 cm × 1 cm × 0.1 cm, were designed to include a surface-active area of 0.2 cm in diameter by drilling a small hole of the same size into the substrate. The substrates were thoroughly cleaned with ethanol and air-dried at room temperature. A solution containing 1 mg mL^−1^ of bare Ag nanoparticles (NPs), Ag/Cu_2_O, or Ag/CuO nanocomposites was prepared and applied to the active surface area of the substrates using a drop-casting method, followed by drying at room temperature. This procedure was repeated for all SERS substrates.

Standard CBZ solutions with concentrations ranging from 10^−3^ to 10^−11^ M were prepared using ethanol as the solvent. For each measurement, 5 μL of a CBZ solution was deposited directly onto the prepared SERS substrates and allowed to evaporate under laboratory conditions. The SERS spectra were recorded immediately afterward using a MacroRaman™ Raman spectrometer (Horiba) equipped with a 785 nm laser excitation. Measurements were performed using a 100× objective with a numerical aperture (NA) of 0.90. The laser power was set to 45 mW, focused at a 45° contact angle, producing a diffraction-limited spot diameter of 1.1 μm (1.22*λ*/NA) and a focal length of 115 nm. Each measurement involved an exposure time of 20 seconds with three accumulations. The final spectrum was obtained after baseline correction.

### 2.4. Electrochemical measurements

Electrochemical measurements in this study were conducted using a Palmsens 4 electrochemical workstation under ambient conditions. The determination of the LUMO energy level of the CBZ analyte followed an established protocol, utilizing a platinum (Pt) working electrode and a silver/silver chloride (Ag/AgCl) reference electrode.^25^ A 0.1 M phosphate-buffered solution (PBS) was used as the electrolyte. All electrochemical potentials were referenced against an internal ferrocene/ferrocenium (Fc/Fc^+^) standard. Cyclic voltammetry (CV) measurements of the CBZ were performed over a potential range of −2 to 2 V at a scan rate of 50 mV s^−1^.

### 2.5. Tea leave sample collection and SERS measurements

Fresh tea leaves (*Camellia sinensis* (L.) Kuntze) were sourced from a local market in Thai Nguyen province, Vietnam. A 100 g portion of the leaves was combined with 300 mL of distilled water, stirred using a vortex mixer, and subjected to ultrasonic treatment for 15 minutes. The resulting mixture was then filtered through filter paper and centrifuged to obtain the supernatant. To prepare CBZ-spiked tea leaf extracts, a precise amount of CBZ standard solution was added to the extract. The spiked extracts were drop-cast onto SERS substrates for subsequent spectral analysis.

## 3. Results and discussion

### 3.1. SERS sensing performance for carbendazim detection using bare Ag nanoparticles

**Fig. 1:**
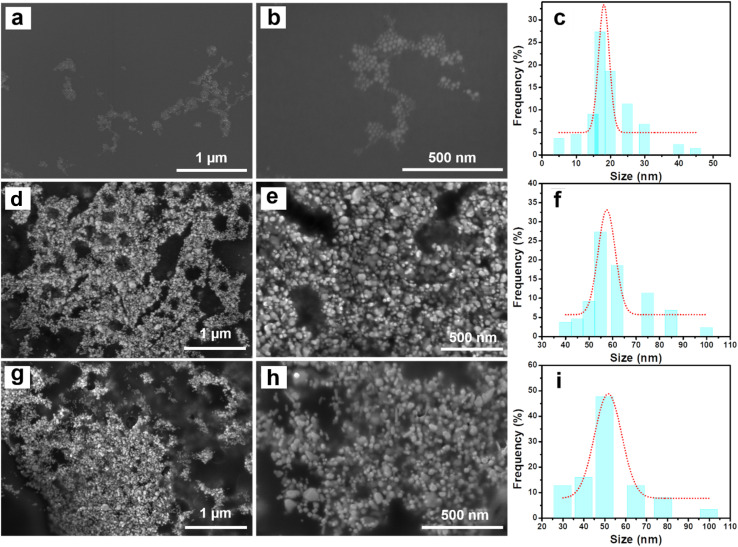
FE-SEM images of Ag NPs (a and b) and Ag/Cu_2_O nanocomposite (d and e) and Ag/CuO nanocomposite (g and h) at different magnification and the size distribution of Ag NPs (c), Cu_2_O (f), CuO (i).

Fig. 1 presents the FE-SEM images of Ag NPs, Ag/Cu_2_O, and Ag/CuO nanocomposites. Fig. 1a and b display the FE-SEM images of Ag NPs, revealing spherical nanoparticles with an average size of approximately 16 nm (see the particle size distribution in Fig. 1c). The FE-SEM images of the Ag/Cu_2_O nanocomposite (Fig. 1d and e) show a dense distribution of spherical nanoparticles. In addition to the spherical particles with an average size of around 16 nm, attributed to Ag NPs, larger spherical particles with an average size of approximately 55 nm are observed, corresponding to Cu_2_O particles (see the particle size distribution in Fig. 1f). A similar phenomenon is noted in the Ag/CuO nanocomposite (Fig. 1g and h), with CuO particles of approximately 50 nm in average size observed (Fig. 1i), demonstrating the uniform dispersion of Cu_2_O and CuO within the Ag/Cu_2_O and Ag/CuO nanocomposites.

**Fig. 2:**
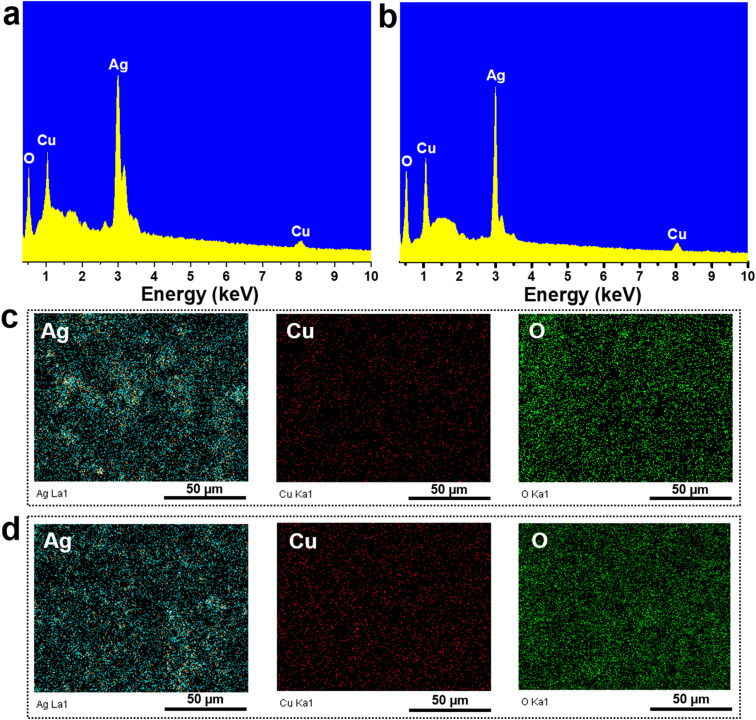
EDX spectra of Ag/Cu_2_O (a) and Ag/CuO (b) nanocomposites; and EDX mapping analysis of Ag/Cu_2_O (c) and Ag/CuO (d) nanocomposites.

**Fig. 3:**
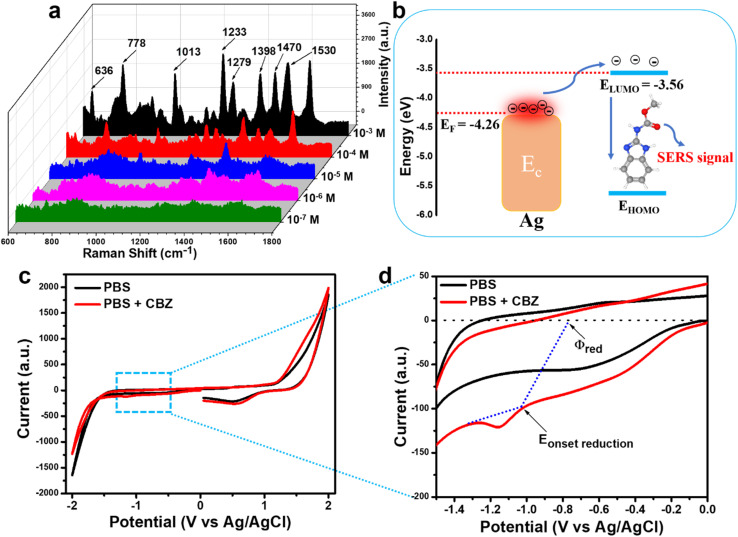
The SERS spectra of CBZ at concentrations ranging from 10^−3^ to 10^−7^ M on bare Ag NPs-based SERS substrates (a), the charge transfer process from Ag NPs to CBZ resulting in enhanced Raman signals (b), and the cyclic voltammetry spectra of CBZ (c and d).

The composition and elemental distribution within the Ag/Cu_2_O and Ag/CuO nanocomposite materials were analyzed using energy-dispersive X-ray spectroscopy (EDX) and elemental mapping (EDX mapping), as presented in Fig. 2. The EDX spectrum in Fig. 2a confirms the presence of silver (Ag), copper (Cu), and oxygen (O) in the Ag/Cu_2_O sample, which correspond well to the expected constituents of Ag and Cu_2_O in the composite. Notably, no extraneous elemental peaks were observed, indicating the high purity of the synthesized material. The EDX elemental mapping (Fig. 2c) reveals a uniform distribution of Cu, O, and Ag throughout the material. Given the relatively low Cu content (10 wt%), copper appears sparsely but evenly distributed, reflecting its actual concentration in the sample. This homogenous spatial distribution of Cu indicates that Cu_2_O is well-dispersed within the Ag matrix of the Ag/Cu_2_O composite. These results validate the successful incorporation and uniform distribution of Cu_2_O in the composite, along with its high purity. Similarly, the EDX spectrum and mapping for the Ag/CuO nanocomposite are shown in Fig. 3b and d. The spectrum confirms the presence of Ag, Cu, and O, with no signals from unintended elements, further verifying the material's purity. The EDX mapping (Fig. 3d) demonstrates a uniform dispersion of Cu and O, suggesting that CuO is evenly distributed within the Ag matrix of the Ag/CuO nanocomposite. Together, these findings confirm the successful formation of both Ag/Cu_2_O and Ag/CuO nanocomposites with high purity and uniform component distribution.

Fig. 3a presents the SERS detection results of CBZ using Ag NPs-based substrates. At a concentration of 10^−3^ M, prominent and well-defined scattering peaks are observed at 636, 778, 1013, 1233, 1279, 1398, 1470, and 1530 cm^−1^, corresponding to characteristic vibrational modes of CBZ. Specifically, the peak at 636 cm^−1^ is attributed to ring stretching and C–C bending vibrations,^26,27^ while the peak at 778 cm^−1^ corresponds to C–H bending in the benzene ring.^27^ The peak at 1013 cm^−1^ is associated with C–C stretching.^26,27^ Peaks at 1233 and 1279 cm^−1^ are attributed to C–H bending vibrations.^26,27^ The peak at 1398 cm^−1^ is assigned to N–H bending,^26,27^ while the peaks at 1470 and 1530 cm^−1^ correspond to C–H bending and C–N stretching vibrations, respectively.^26,27^ As the CBZ concentration decreases to 10^−4^, 10^−5^, and 10^−6^ M, the intensity of these characteristic peaks gradually diminishes. At a concentration of 10^−7^ M, the characteristic peaks of CBZ become indistinguishable, establishing the detection limit of the Ag NPs substrate at 10^−6^ M. This detection limit is clearly insufficient to meet the sensitivity expectations of a platform as sensitive as SERS.

The alignment of energy levels between the Fermi level (*E*_F_) of Ag and the lowest unoccupied molecular orbital (LUMO) of the analyte molecule is critical and significantly influences the overall SERS signal obtained.^28^ We calculated the LUMO energy level of CBZ based on cyclic voltammetry (CV) measurements. The onset reduction potential (*ϕ*_red_) was determined to calculate the LUMO energy level (*E*_LUMO_) of CBZ using the following formula:^25,29,30^*E*_LUMO_ = −*e*(*ϕ*_red_ + 4.8 − *ϕ*_Fc/Fc_^+^) here, *ϕ*_Fc/Fc^+^_ represents the redox potential of the ferrocene/ferrocenium (Fc/Fc^+^) couple in the electrochemical measurement system, with the energy level of Fc/Fc^+^ assumed to be −4.8 eV relative to the vacuum level. For this study, we used an electrochemical setup similar to that described by Bin *et al.*, employing a Pt working electrode and an Ag/AgCl reference electrode.^25^ Consequently, *ϕ*_Fc/Fc^+^_ was assumed to be 0.44 V *versus* Ag/AgCl. The CV results are presented in Fig. 3c and d. The *ϕ*_red_ of CBZ was found to be −0.80 V, yielding a calculated ELUMO of −3.56 eV. The energy difference between the *E*_F_ level of Ag NPs and the LUMO of CBZ is relatively large (0.7 eV), creating a significant energy barrier for charge transfer from Ag to CBZ. This results in challenges in the charge transfer process, leading to a weaker SERS enhancement, as shown in Fig. 3b.

### 3.2. Comparison of SERS sensor performance for carbendazim detection on Ag/Cu_2_O and Ag/CuO nanocomposites

**Fig. 4:**
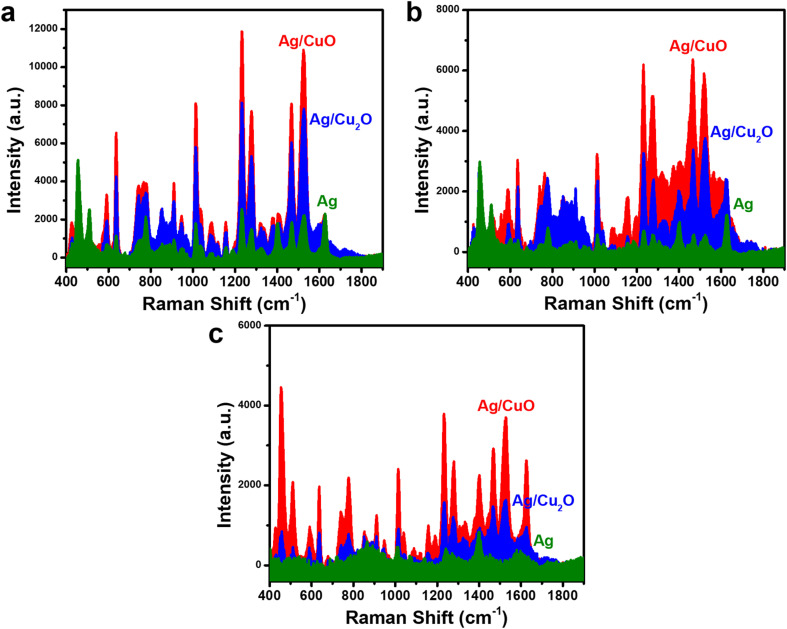
Comparison of SERS spectra for the detection of CBZ using bare Ag NPs, Ag/Cu_2_O, and Ag/CuO nanocomposites at concentrations of 10^−3^ M (a), 10^−4^ M (b), and 10^−5^ M (c).

To improve the SERS sensor performance for CBZ detection, two Ag-based nanocomposite materials, Ag/Cu_2_O and Ag/CuO, were employed to detect CBZ at concentrations of 10^−3^, 10^−4^, and 10^−5^ M, and compared with bare Ag NPs. The results are presented in Fig. 4. Fig. 4a shows the SERS spectra of CBZ at a concentration of 10^−3^ M for all three materials: bare Ag NPs, Ag/Cu_2_O, and Ag/CuO. It is evident that both nanocomposites enhance the SERS signal of CBZ more effectively than bare Ag NPs, with the Ag/CuO nanocomposite demonstrating the strongest enhancement. This trend is consistently observed at lower concentrations of 10^−4^ M (Figure 4b) and 10^−5^ M (Fig. 4c). Therefore, the incorporation of semiconducting materials Cu_2_O and CuO into the nanocomposites significantly improves the overall SERS signal for CBZ. Additionally, the enhancement efficiency differs between the Ag/Cu_2_O and Ag/CuO composites, with bare Ag NPs exhibiting the lowest SERS efficiency, followed by Ag/Cu_2_O, which offers a higher enhancement than bare Ag NPs but less than Ag/CuO. This variation in SERS enhancement across the three materials suggests a complex enhancement mechanism, driven by the interaction between Ag, Cu_2_O (or CuO), and CBZ. This indicates that while Ag alone provides EM enhancement *via* surface plasmon resonance, it is not sufficient to achieve optimal SERS performance. The better performance of Ag/CuO and Ag/Cu_2_O nanocomposites, in particular, suggests that the charge transfer-based CM enhancement mechanism – enabled by the semiconductor component – plays an important role alongside EM. Therefore, the enhanced SERS effect arises from a synergistic interaction between the EM contribution of Ag and the CM (charge transfer) contribution of CuO and Cu_2_O rather than from EM alone, underscoring the importance of combining both mechanisms for optimal SERS sensitivity.

**Fig. 5:**
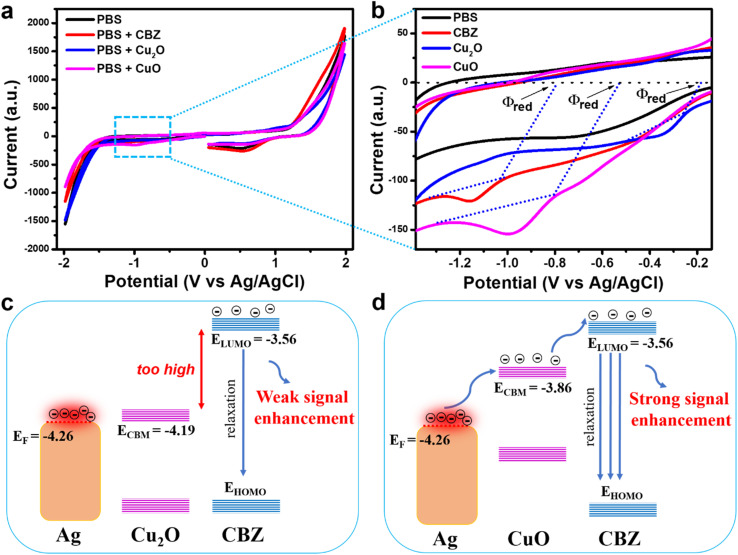
Cyclic voltammetry of Cu_2_O and CuO (a and b) and the energy level alignment of the components Ag, Cu_2_O (c), CuO (d), and CBZ in the SERS signal enhancement mechanism.

While EM enhancement due to the surface plasmon resonance of Ag is a well-known mechanism in SERS, in this system it alone is insufficient to produce a strong SERS response, as evidenced by the weaker signals from bare Ag NPs. This indicates that EM enhancement from Ag, though present, does not dominate the overall SERS activity. As analyzed in Section 3.1, the alignment between the *E*_F_ level of Ag and the LUMO energy level of CBZ plays a critical role in the overall signal enhancement. The weak SERS signal from bare Ag NPs arises due to the large energy gap between *E*_F_ level of Ag and the LUMO level of CBZ, which hinders the charge transfer from Ag to CBZ (resulting in a SERS signal without contribution from the CM mechanism). The enhanced SERS signal in the appropriate presence of Cu_2_O and CuO in the nanocomposite structure may be due to their facilitation of charge transfer. CV measurements were performed to determine the minimum conduction band (*E*_CBM_) energy levels of Cu_2_O and CuO, with results shown in Fig. 5a and b. Based on the determined onset reduction potentials (*ϕ*_red_) of Cu_2_O and CuO, which were −0.17 V and −0.54 V, respectively, the ECBM values for Cu_2_O and CuO were calculated to be −4.19 eV and −3.86 eV. Fig. 5c and d illustrate the alignment of the energy levels of Ag, Cu_2_O, and CBZ in the Ag/Cu_2_O nanocomposite, and Ag, CuO, and CBZ in the Ag/CuO nanocomposite. It can be observed that the *E*_CBM_ of both Cu_2_O and CuO lies between the *E*_F_ of Ag and the LUMO of CBZ, potentially facilitating charge transfer from Ag to CBZ by reducing the potential energy barrier between Ag and CBZ. Interestingly, the *E*_CBM_ of Cu_2_O (−4.19 eV) is close to the *E*_F_ of Ag (−4.26 eV), which makes the reduction of the energy barrier less effective. In contrast, the *E*_CBM_ of CuO (−3.86 eV) is well-positioned between the *E*_F_ of Ag and the *E*_LUMO_ of CBZ, providing the optimal condition for charge transfer from Ag to CBZ *via* this energy bridge. This observation aligns with the trend of the SERS signals obtained from the three materials: bare Ag NPs showing the lowest SERS efficiency, followed by Ag/Cu_2_O, and the highest with Ag/CuO. These findings highlight the crucial role of the alignment of energy levels of the components in the nanocomposite material with the analyte in determining the overall SERS signal enhancement. These results suggest that while EM effects originating from Ag contribute to the SERS activity, they are not the dominant mechanism in this case. Instead, the markedly improved performance of Ag/CuO underscores the importance of combining EM with an efficient CM enhancement pathway, realized through the favorable energy level alignment that facilitates interfacial charge transfer. This synergy between EM and CM is critical for achieving optimal SERS sensitivity in such hybrid nanostructures. With the highest SERS signal enhancement, the Ag/CuO nanocomposite was selected for evaluating the sensor's performance for CBZ detection.

### 3.3. SERS sensing performance for carbendazim detection using Ag/CuO nanocomposite

**Fig. 6:**
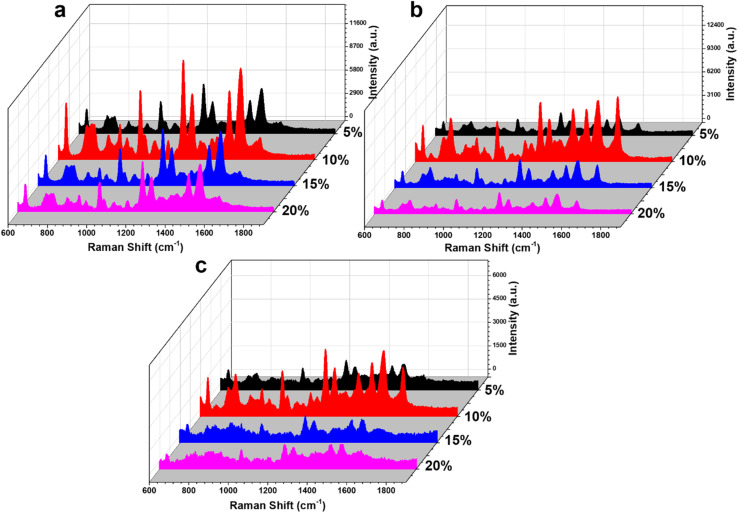
The SERS spectra of CBZ on Ag/CuO nanocomposite substrates with varying CuO content at concentrations of 10^−3^ M (a), 10^−4^ M (b), and 10^−5^ M (c).

The SERS spectra of CBZ on Ag/CuO nanocomposites with varying CuO weight percentages (5%, 10%, 15%, and 20%) were analyzed to determine the optimal CuO content for signal enhancement. Fig. 6 displays the SERS results of CBZ at concentrations of 10^−3^, 10^−4^, and 10^−5^ M for the Ag/CuO nanocomposites with different CuO loadings. The results clearly show that the CuO content significantly influences the overall SERS enhancement. At a concentration of 10^−3^ M (Fig. 6a), the Ag/CuO sample with 10% CuO by weight exhibited the highest SERS signal for CBZ. This trend was consistent at the lower CBZ concentrations of 10^−4^ and 10^−5^ M as well (Fig. 6b and c). This optimal CuO content implies an achieved balance between the EM enhancement contributed by the Ag component and the CM enhancement derived from the CuO component, resulting in the highest overall SERS signal. The appropriate presence of CuO (10%) provides favorable interfacial interaction sites that facilitate charge transfer from Ag through the CuO bridge to the analyte, thereby enhancing the CT efficiency while preserving sufficient plasmonic Ag content, thus maintaining strong EM enhancement. In contrast, lower CuO content (*e.g.*, 5%) may offer insufficient semiconductor surface for effective CT, while higher contents (15–20%) may lead to excessive coverage of the Ag surface, diminishing plasmonic activity. Therefore, 10% CuO achieves a synergistic balance between both SERS enhancement mechanisms. Based on these findings, the Ag/CuO nanocomposite with 10% CuO content was selected for further evaluation of its sensor performance, including sensitivity, reliability, and practicality.

**Fig. 7:**
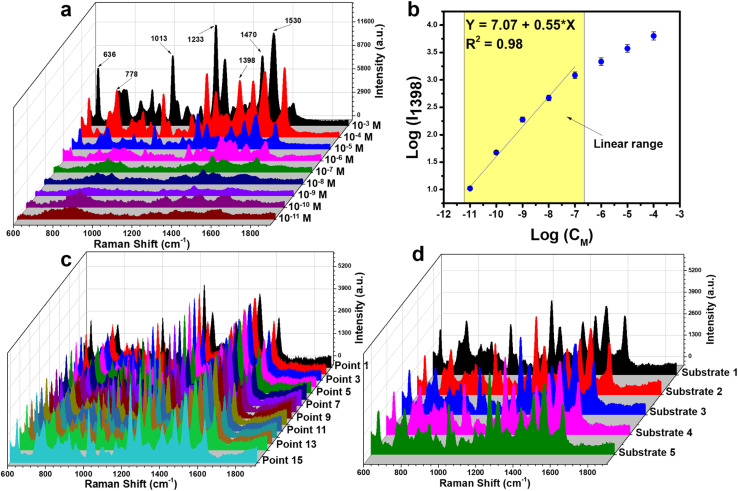
SERS spectra of CBZ at concentrations ranging from 10^−3^ to 10^−11^ M on Ag/CuO nanocomposite substrate (a), logarithmic plots of intensity and CBZ concentration at 1398 cm^−1^ (b), reliability assessment of the substrate through measurements at 15 points on the same substrate (c) and across 5 different substrates (d).

Fig. 7a presents the SERS spectra of CBZ collected on Ag/CuO nanocomposite substrates across a concentration range of 10^−3^ to 10^−11^ M. As shown, at higher concentrations of 10^−3^, 10^−4^, and 10^−5^ M, the characteristic peaks of CBZ are clearly visible. These peaks gradually diminish as the concentration decreases. Notably, at the lower concentrations of 10^−9^ and 10^−10^ M, the characteristic peaks of CBZ are still detectable, with the peaks disappearing entirely only at a concentration of 10^−11^ M. A linear relationship between CBZ concentration and SERS intensity was calculated to determine the linear equation. Four peaks at 1013 cm^−1^, 1233 cm^−1^, 1398 cm^−1^, and 1530 cm^−1^ were used to define this relationship based on a logarithmic function. The results are shown in Fig. 7b and S1.† The 1398 cm^−1^ peak demonstrated the best linearity with a correlation coefficient of 0.98, while the other peaks had correlation coefficients below 0.90. The linear range was determined to be between 10^−7^ M and 10^−11^ M, and the linear equation was found to be *y* = 7.07 + 0.55 × *x*, where *x* and *y* represent the logarithmic functions of CBZ concentration and intensity, respectively. Based on this equation, the limit of detection (LOD) for CBZ using Ag/CuO nanocomposite was calculated to be 8.85 × 10^−11^ M (further details of calculation are provided in ESI†). With such high sensitivity, the enhancement factor (EF) of Ag/CuO nanocomposite was calculated for a 10^−10^ M concentration at the 1398 cm^−1^ peak, yielding a result of 7.81 × 10^7^ (detailed calculation information is also provided in ESI†). Table 1 compares the SERS sensor performance for detecting CBZ with Ag/CuO nanocomposites in this study to recent research. The sensitivity of the Ag/CuO nanocomposite-based SERS substrate is superior to other substrates, as indicated by its impressive LOD value, surpassing other substrates. Previous SERS substrates established a detection limit of 10^−9^ M, while the Ag/CuO nanocomposite can detect CBZ as low as 10^−11^ M. Notably, the three-dimensional (3D) cactus-like Ag/CuO/Cu_2_O nanocomposite structure – comprising a complex and sophisticated SERS substrate – has previously demonstrated a good detection limit for CBZ at 1.5 × 10^−10^ M. However, the Ag/CuO nanocomposite substrate developed in this study exhibits even better CBZ detection performance.

**Table 1:** Evaluate the sensing performance of CBZ detection using Ag/CuO nanocomposite substrate with recent studies

Substrate	LOD	*E*_F_	Ref.
Au nanorods	5.0 × 10^−5^ M	—	31
Ag/SiO_2_ nanocomposite	5.2 × 10^−7^ M	—	32
Ag NPs decorated ZnO nanorods	5.2 × 10^−9^ M	—	33
Ag@SiO_2_-molecularly imprinted polymer composite	1.0 × 10^−9^ M	—	34
3D cactus-like Ag/CuO/Cu_2_O nanocomposite	1.5 × 10^−10^ M	—	35
Ag/CuO nanocomposite	8.85 × 10^−11^ M	7.81 × 10^7^	This work

**Fig. 8:**
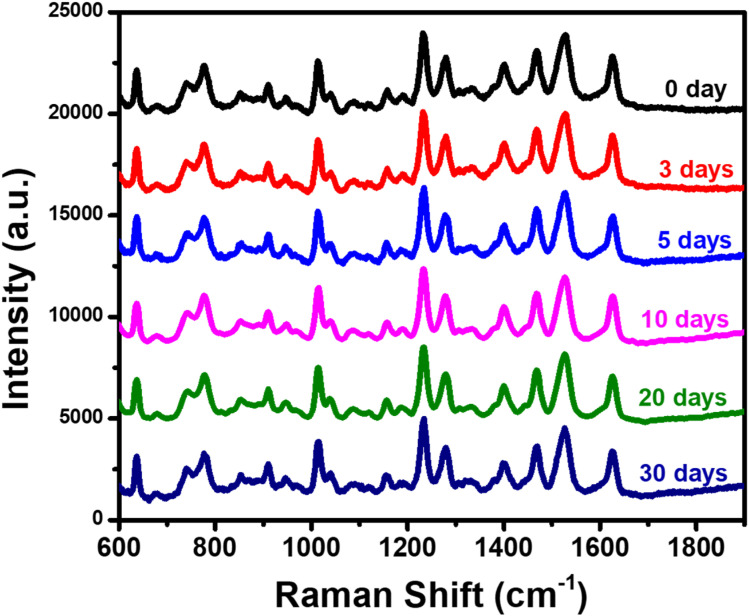
Stability of the Ag/TiO_2_ nanocomposite SERS substrate evaluated by monitoring SERS spectra collected at different storage time points.

The reliability of the Ag/CuO nanocomposite-based SERS substrate was evaluated *via* two parameters: repeatability and reproducibility. The results are presented in Fig. 7c and d. Repeatability was assessed by collecting SERS signals of CBZ at a concentration of 10^−5^ M at five different points on the same substrate (Fig. 7c). It can be observed that the characteristic peaks of CBZ appear uniformly across all fifteen spectra, both in terms of position and peak intensity. Quantitatively, the relative standard deviation (RSD) was calculated, yielding a result of 5.32%, demonstrating high repeatability of the Ag/CuO nanocomposite substrate. Reproducibility was evaluated by preparing 5 different SERS substrates and collecting SERS spectra from these five substrates at a CBZ concentration of 10^−5^ M (Fig. 7d). The RSD for reproducibility was calculated to be 8.22%, indicating high reproducibility of the Ag/CuO nanocomposite-based SERS substrate. With both RSD values being less than 10% for repeatability and reproducibility, the high reliability of the Ag/CuO nanocomposite substrate is confirmed. The stability of the SERS substrate based on Ag/CuO nanocomposites was also evaluated by monitoring its ability to retain SERS signal intensity over various storage time points. The substrates were stored in a sealed box to prevent direct exposure to light. The results are shown in Fig. 8. It can be observed that the SERS spectra collected at different storage durations closely resemble the initial spectrum (day 0) obtained from 10^−5^ M of CBZ, both in terms of signal intensity and the presence of characteristic peaks. No peak disappearance or emergence of new scattering peaks was detected during the 30-day storage period. Moreover, the SERS intensity remained consistently high at the 3, 5, 10, 20, and 30-day marks, indicating that the Ag/CuO nanocomposite substrate possesses high long-term stability.

### 3.4. SERS sensor performance for detecting carbendazim in tea leaves based on Ag/CuO nanocomposite substrate

**Fig. 9:**
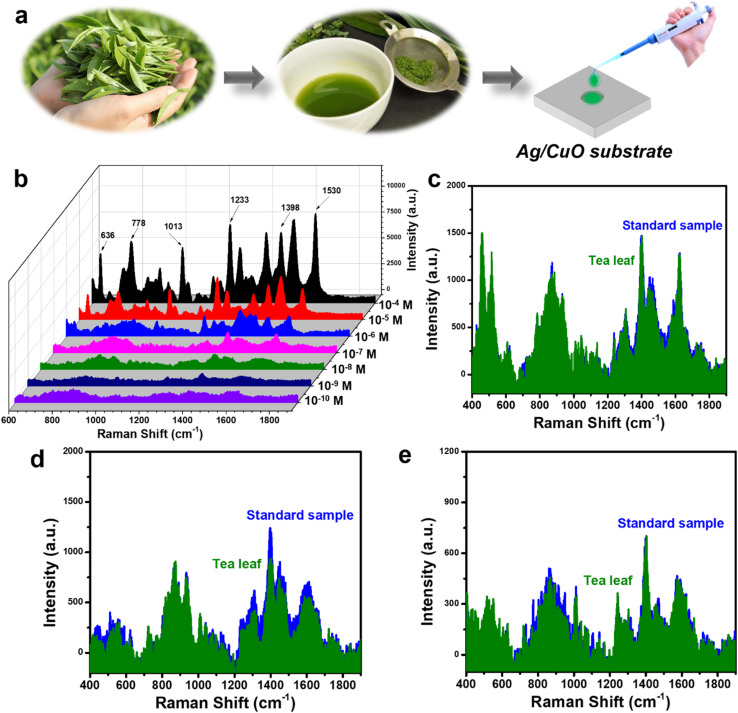
Preparation of Ag/CuO substrate for collecting SERS signals on tea leaves (a), SERS spectra of CBZ in tea leaf samples at concentrations ranging from 10^−4^ to 10^−10^ M (b), and comparison of SERS signals of CBZ in tea leaf samples with standard solutions at concentrations of 10^−7^ M (c), 10^−8^ M (d), and 10^−9^ M (e).

The practicability of the Ag/CuO nanocomposite substrate was assessed by its ability to detect CBZ in fresh tea leaf samples. The sample preparation procedure for fresh tea leaves is described in Section 2.5 and as shown in Fig. 9a. Fresh tea leaf samples containing CBZ at concentrations ranging from 10^−4^ to 10^−10^ M were applied to the Ag/CuO SERS substrate, and SERS signals were collected from these substrates. The results are presented in Fig. 9b. It can be seen that the characteristic peaks of CBZ are clearly visible at concentrations of 10^−4^, 10^−5^, 10^−6^, and 10^−7^ M. At a concentration of 10^−9^ M, the characteristic peak at 1398 cm^−1^ for CBZ is still present. Fig. 9c–e compare the SERS signals obtained from the fresh tea leaf samples with the SERS signals from a standard solution sample at concentrations of 10^−7^, 10^−8^, and 10^−9^ M, respectively. A high degree of overlap can be observed between the characteristic peaks for CBZ obtained from the two samples. The recovery values were calculated and are shown in Table 2, ranging from 85% to 106%, indicating high practicability. Therefore, the Ag/CuO nanocomposite-based SERS substrate, in addition to its high sensitivity and reliability, also demonstrates high practicability in the analysis of field samples.

**Table 2:** CBZ concentration detected by Ag/CuO nanocomposite-based SERS sensor in tea leaf, along with their calculated recovery values

Samples	Spiked (M)	Detected at 1398 cm^−1^ (M)	Recovery at 1398 cm^−1^ (%)	Detected at 1627 cm^−1^ (M)	Recovery at 1627 cm^−1^ (%)
Tea leaves	10^−4^	9.04 × 10^−5^	90	9.25 × 10^−5^	92
	10^−5^	8.74 × 10^−6^	87	9.11 × 10^−6^	91
	10^−6^	1.06 × 10^−6^	106	9.41 × 10^−7^	94
	10^−7^	9.83 × 10^−8^	98	9.91 × 10^−8^	99
	10^−8^	8.51 × 10^−9^	85	8.72 × 10^−9^	87
	10^−9^	1.02 × 10^−9^	102	9.88 × 10^−10^	99

## 4. Conclusions

In this study, we successfully developed a sensitive, reliable, and practical Ag/CuO nanocomposite SERS substrate through a simple green electrochemical method for detecting carbendazim residues in tea leaves. The substrate can detect carbendazim at an ultra-low limit of 8.85 × 10^−11^ M, which highlights its potential for ultrasensitive pesticide detection. The substrate also showed excellent repeatability and reproducibility with an RSD of less than 10%, and its practicability was validated by the accurate detection of CBZ in fresh tea leaves, achieving recovery values ranging from 85% to 106%. Furthermore, the exploration of the SERS enhancement mechanism revealed the critical role of metal/semiconductor transitions in boosting sensing performance, offering valuable insights for designing advanced SERS substrates. These findings demonstrate that the Ag/CuO nanocomposite is a promising candidate for practical agricultural and environmental monitoring, offering a versatile, field-ready platform for SERS applications.

## Data availability

The data that support the findings of this study are available from the corresponding author upon reasonable request. All experimental data, including the characterization of the Ag/CuO and Ag/Cu_2_O nanocomposites, and the detection results of carbendazim pesticide, are included within the manuscript and its ESI.†

## Author contributions

D. T. Linh: investigation, formal analysis, writing – original draft; Q. D. Mai: conceptualization, methodology, investigation, formal analysis, writing – original draft; D. T. H. Trang: validation, investigation; N. T. Anh: conceptualization, methodology, investigation; A. T. Le: conceptualization, methodology, supervision, project administration, writing – review & editing.

## Conflicts of interest

The authors declare that they have no known competing financial interests or personal relationships that could have appeared to influence the work reported in this paper.

## Supplementary Material

RA-015-D5RA00846H-s001

## References

[cit1] Han X. X., Rodriguez R. S., Haynes C. L., Ozaki Y., Zhao B. (2021). Surface-enhanced Raman spectroscopy. Nat. Rev. Methods Primers.

[cit2] Langer J., Jimenez de Aberasturi D., Aizpurua J., Alvarez-Puebla R. A., Auguié B., Baumberg J. J., Bazan G. C., Bell S. E., Boisen A., Brolo A. G. (2019). Present and future of surface-enhanced Raman scattering. ACS Nano.

[cit3] Sharma B., Frontiera R. R., Henry A.-I., Ringe E., Van Duyne R. P. (2012). SERS: Materials, applications, and the future. Mater. Today.

[cit4] Nie S., Emory S. R. (1997). Probing single molecules and single nanoparticles by surface-enhanced Raman scattering. Science.

[cit5] Li H., Dumont E., Slipets R., Thersleff T., Boisen A., Sotiriou G. A. (2023). Democratizing robust SERS nano-sensors for food safety diagnostics. Chem. Eng. J..

[cit6] Liu C., Xu D., Dong X., Huang Q. (2022). A review: Research progress of SERS-based sensors for agricultural applications. Trends Food Sci. Technol..

[cit7] Ding S.-Y., Yi J., Li J.-F., Ren B., Wu D.-Y., Panneerselvam R., Tian Z.-Q. (2016). Nanostructure-based plasmon-enhanced Raman spectroscopy for surface analysis of materials. Nat. Rev. Mater..

[cit8] Graham D., Moskovits M., Tian Z.-Q. (2017). SERS–facts, figures and the future. Chem. Soc. Rev..

[cit9] Ding S.-Y., You E.-M., Tian Z.-Q., Moskovits M. (2017). Electromagnetic theories of surface-enhanced Raman spectroscopy. Chem. Soc. Rev..

[cit10] Chen R., Jensen L. (2023). Interpreting chemical enhancements of surface-enhanced Raman scattering. Chem. Phys. Rev..

[cit11] Ying Y., Tang Z., Liu Y. (2023). Material design, development, and trend for surface-enhanced Raman scattering substrates. Nanoscale.

[cit12] Liu Y., Ma H., Han X. X., Zhao B. (2021). Metal–semiconductor heterostructures for surface-enhanced Raman scattering: synergistic contribution of plasmons and charge transfer. Mater. Horiz..

[cit13] Procházka M., Novák D., Kočišová E., Kylián O. e., Ji W., Ozaki Y. (2024). New Insights into SERS Mechanism of Semiconductor–Metal Heterostructure: A Case Study on Vanadium Pentoxide Nanoparticles Decorated with Gold. J. Phys. Chem. C.

[cit14] Wang Q., Chang K., Yang Q., Wu W. (2024). Semiconductor-based surface-enhanced Raman scattering sensing platforms: State of the art, applications and prospects in food safety. Trends Food Sci. Technol..

[cit15] Huang Y., Zhang S., Jiang S., Xu J. (2024). Improved SERS Performance on Ag-Coated Amorphous TiO2 Random Nanocavities by the Enhanced Light–Matter Coupling Effect. ACS Sustain. Chem. Eng..

[cit16] Liu L., Yang H., Ren X., Tang J., Li Y., Zhang X., Cheng Z. (2015). Au–ZnO hybrid nanoparticles exhibiting strong charge-transfer-induced SERS for recyclable SERS-active substrates. Nanoscale.

[cit17] Mai Q.-D., Thanh D. C., Anh N. T., Van Manh T., Bach T. N., Nguyen H.-A., Pham A.-T., Le A.-T. (2024). Smart 3D Ag-decorated TiO2 Nanostructure: An Advanced Synergistic SERS Substrate for Trace Detection of Analytes with Diverse Natures. Sens. Actuators, B.

[cit18] Huang S., Wu C., Wang Y., Yang X., Yuan R., Chai Y. (2021). Ag/TiO2 nanocomposites as a novel SERS substrate for construction of sensitive biosensor. Sens. Actuators, B.

[cit19] Xue X., Zhao C., Qiao Y., Wang P., Wang J., Shi J., Liu B., Wang Z., Hou E., Chang L. (2024). A novel three-dimensional porous Ag/TiO2 hybrid aerogels with high dense hot spot as effective SERS substrate for ultrasensitive detection. Spectrochim. Acta, Part A.

[cit20] Yang J., Song G., Zhou L., Wang X., You L., Li J. (2021). Highly sensitively detecting tetramethylthiuram disulfide based on synergistic contribution of metal and semiconductor in stable Ag/TiO2 core-shell SERS substrates. Appl. Surf. Sci..

[cit21] Pal A. K., Pagal S., Prashanth K., Chandra G. K., Umapathy S. (2019). Ag/ZnO/Au 3D hybrid structured reusable SERS substrate as highly sensitive platform for DNA detection. Sens. Actuators, B.

[cit22] Yang B., Shao X., Gu X., Wang K., Ning X., Xia J., Xie M., Tang Y., Li Q., Tian S. (2023). CuO@ AgNPs nanozyme cavity arrays on screen-printed electrodes for ultrasensitive and on-site SERS detection. Chem. Eng. J..

[cit23] Linh D. T., Mai Q.-D., Nga D. T. N., Anh N. T., Van Tuan H., Nguyen H. A., Vu X. H., Le A.-T. (2024). Surface ligand modified silver nanoparticles-based SERS sensing platform for ultrasensitive detection of the pesticide thiram in green tea leaves: roles of coating agents in sensing performance. RSC Adv..

[cit24] Anh N. T., Dinh N. X., Pham T. N., Le A.-T. (2021). Enhancing the chloramphenicol sensing performance of Cu–MoS 2 nanocomposite-based electrochemical nanosensors: roles of phase composition and copper loading amount. RSC Adv..

[cit25] Bin H., Gao L., Zhang Z.-G., Yang Y., Zhang Y., Zhang C., Chen S., Xue L., Yang C., Xiao M. (2016). 11.4% Efficiency non-fullerene polymer solar cells with trialkylsilyl substituted 2D-conjugated polymer as donor. Nat. Commun..

[cit26] Furini L. N., Sanchez-Cortes S., López-Tocón I., Otero J. C., Aroca R. F., Constantino C. J. L. (2015). Detection and quantitative analysis of carbendazim herbicide on Ag nanoparticles via surface-enhanced Raman scattering. J. Raman Spectrosc..

[cit27] He J., Li H., Zhang L., Zhi X., Li X., Wang X., Feng Z., Shen G., Ding X. (2021). Silver microspheres aggregation-induced Raman enhanced scattering used for rapid detection of carbendazim in Chinese tea. Food Chem..

[cit28] Mai Q. D., Nguyen H. A., Phung T. L. H., Xuan Dinh N., Tran Q. H., Doan T. Q., Le A.-T. (2022). Silver nanoparticles-based SERS platform towards detecting chloramphenicol and amoxicillin: an experimental insight into the role of HOMO–LUMO energy levels of the analyte in the SERS signal and charge transfer process. J. Phys. Chem. C.

[cit29] Sun Q., Wang H., Yang C., Li Y. (2003). Synthesis and electroluminescence of novel copolymers containing crown ether spacers. J. Mater. Chem..

[cit30] Eckhardt H., Shacklette L., Jen K., Elsenbaumer R. (1989). The electronic and electrochemical properties of poly (phenylene vinylenes) and poly (thienylene vinylenes): An experimental and theoretical study. J. Chem. Phys..

[cit31] Strickland A. D., Batt C. A. (2009). Detection of carbendazim by surface-enhanced Raman scattering using cyclodextrin inclusion complexes on gold nanorods. Anal. Chem..

[cit32] Thien N. D., Hoa N. Q., Tu N. N., Doanh S. C., Long N. N., Vu L. V. (2019). Detection of carbendazim by SERS technique using silver nanoparticles decorated SiO 2 opal crystal substrates. J. Electron. Mater..

[cit33] Luong H. N., Nguyen N. M., Tran C. K., Nguyen T. T., Nguyen N. P., Huynh T. M. H., Tran T. T., Phan B. T., Thi T. V. T., Dang V. Q. (2022). Detection of carbendazim by utilizing multi-shaped Ag NPs decorated ZnO NRs on patterned stretchable substrate through surface-enhanced Raman scattering effect. Sens. Actuators, A.

[cit34] Cheshari E. C., Ren X., Li X. (2020). Core–shell Ag-molecularly imprinted composite for SERS detection of carbendazim. Int. J. Environ. Anal. Chem..

[cit35] Lu Y., Bi Z., Shang G. (2022). Facile method to fabricate cactus-like Ag NPs/CuO/Cu2O nanocomposites for recyclable SERS detection of trace carbendazim residues. ACS Appl. Nano Mater..

