# Supplementary material for: Ultrasensitive detection of carbendazim pesticide in tea leaves using a green Ag/CuO(Cu2O) nanocomposite-based SERS sensor: role of metal/semiconductor transition in sensing performance
Source: RSC Adv. 2025 May 27;15(22):17635–47. doi: 10.1039/d5ra00846h (PMC12108759; doi:10.1039/d5ra00846h)
Supplement: RA-015-D5RA00846H-s001 [file RA-015-D5RA00846H-s001.pdf]

Supplementary Information (SI) for RSC Advances.
This journal is © The Royal Society of Chemistry 2025

Supporting Information

Ultrasensitive Detection of Carbendazim Pesticide in Tea Leaves using Green Ag/CuO(Cu_2O) Nanocomposite-based SERS Sensor: Role of Metal/Semiconductor Transition in Sensing Performance

Dong Thi Linh[a,b], Quan-Doan Mai[a,*], Dang Thi Hanh Trang[a], Nguyen Tuan Anh[a], Xuan Hoa Vu[c], Anh-Tuan Le[a,d,**]

[a]*Phenikaa University Nano Institute (PHENA), Phenikaa University, Hanoi 12116, Vietnam*

[b] *Faculty of Fundamental Sciences, Thai Nguyen University of Technology, 666 3/2 road, Thai Nguyen city 24000, Vietnam*

[c]*Institute of Science and Technology, TNU-University of Sciences, Tan Thinh ward, Thai Nguyen city 24000, Vietnam*

[d]*Faculty of Materials Science and Engineering (MSE), Phenikaa University, Hanoi 12116, Vietnam*

Corresponding authors:

*doan.maiquan@phenikaa-uni.edu.vn (Q.D. Mai)

**tuan.leanh@phenikaa-uni.edu.vn (A.T. Le)

Calculation of limit of detection (LOD)

The standard curve of linear detecting range was given as:

$$Y = A + B \times Log(X) \tag{1}$$

where A and B are intercept and slope of regression equation obtained through the plot of the logarithmic SERS intensity (Y) – logarithmic concentration (X).

The LOD is calculated using the following equation[1]:

$$LOD = 10^{\left[\left(Y_{blank}\ +\ 3SD\right)/Y_{blank} - A\right]/B} \tag{2}$$

where Y_{blank} and SD are the SERS signal and the standard deviation of blank sample, respectively.

SD is calculated via the well-known formula:

$$SD = \sqrt{\frac{1}{n-1} \times \sum_{i}^{n} (x_i - x_{average})^2} \tag{3}$$

where x_i if the "i" sample of the series of measurements, $x_{average}$ is the average value of SERS signal obtained from the blank sample repeated n times.

Calculation of enhancement factor (EF)

The EF value is calculated according to the well-established equation, which was employed in several published studies[2,3]:

$$EF = \frac{I_{SERS}}{I_{Raman}} \times \frac{N_{bulk}}{N_{surface}} \tag{4}$$

where I_{SERS} and I_{Raman} are Raman signal intensity of the analyte with and without SERS from the substrate, respectively; and N_{bulk} is the number of analyte molecules that are probed on the Raman spectrum, while $N_{surface}$ is the number of analyte molecules probed using SERS.

N_{bulk} can be calculated following:

$$N_{bulk} = \frac{A_{laser} \times h \times \rho}{M} \times N_A \quad (5)$$

where A_{laser}, h, ρ and m are the laser spot area, the focal length, the density of the solid analyte and its molecular weight, respectively; and N_A is the Avogadro number.

$N_{surface}$ can be expressed as:

$$N_{surface} = \frac{C \times V}{A_{substrate}} \times N_A \times A_{laser} \quad (6)$$

where C, V, $A_{substrate}$ are the concentration, the volume drop-casted of the analyte, and the area of the substrate, respectively; N_A is the Avogadro number; and A_{laser} is the laser spot area.

Thus EF can be calculated as:

$$EF = \frac{I_{SERS}}{I_{Raman}} \times \frac{N_{bulk}}{N_{surface}} = \frac{I_{SERS}}{I_{Raman}} \times \frac{h \times \rho \times A_{substrate}}{M \times C \times V} \quad (7)$$

In our case, I_{Raman} is Raman signal intensity without SERS substrate of carbendazim, h = 2 µm, $\rho_{carbendazim} = 1.45$ g/cm^3; $M_{carbendazim} = 191$ g/mol; $A_{substrate} = 4$ mm^2, V = 5 µL.

Calculation of relative standard deviation (RSD)

The RSD value of repeatability and reproducibility is calculated via the well-known formula:

$$\text{RSD} = \frac{SD \times 100}{x_{average}} \tag{8}$$

where SD is the standard deviation that calculates using equation 3 and $x_{average}$ is the average value of SERS signal obtained from each measurement.

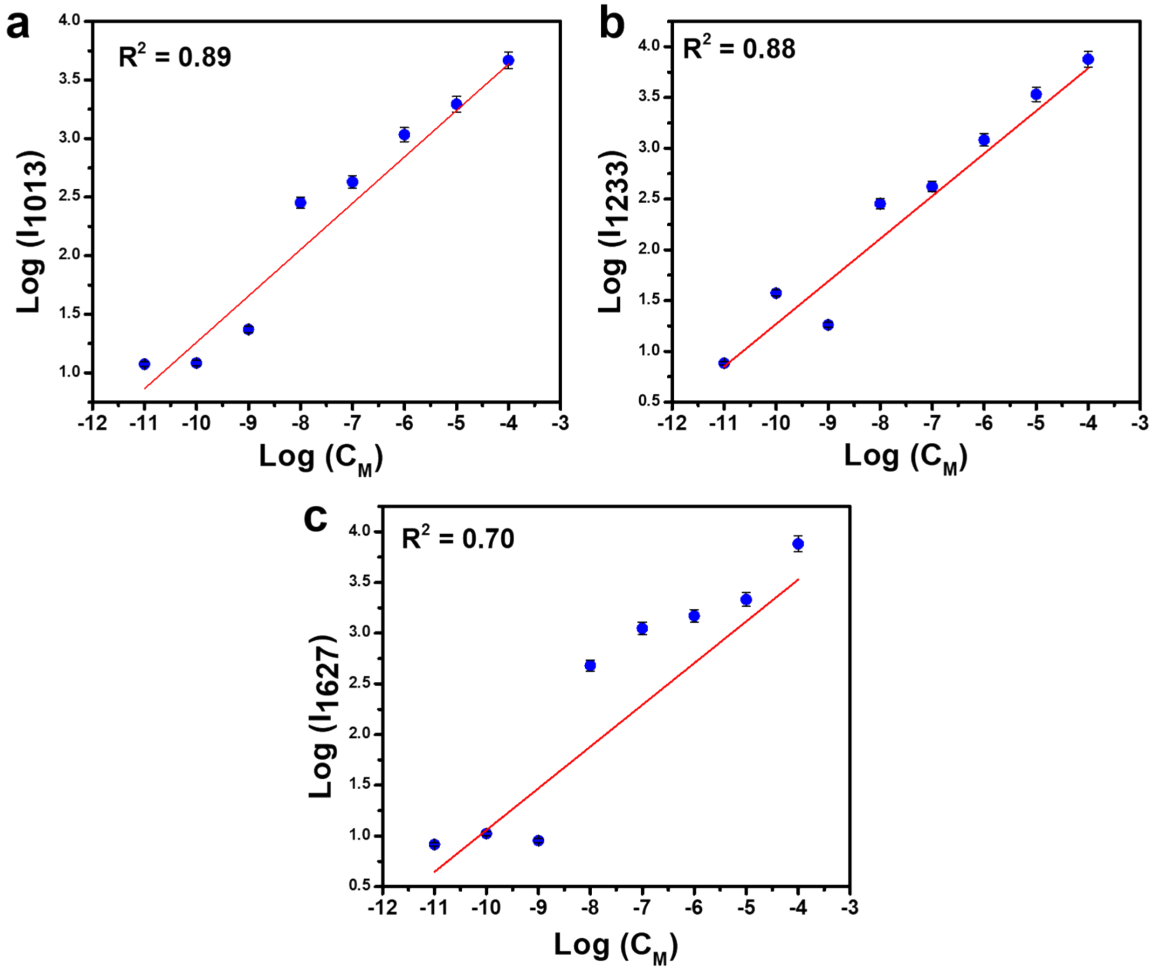

Figure S1. Logarithmic plots of SERS intensity versus CBZ concentration at 1013 cm^{-1} (a), 1233 cm^{-1} (b) and 1627 cm^{-1} (c).

References

1 Chen, R. *et al.* Dual-amplification strategy-based SERS chip for sensitive and reproducible detection of DNA methyltransferase activity in human serum. *Analytical chemistry* **91**, 3597-3603 (2019).

2 Le Ru, E. C., Blackie, E., Meyer, M. & Etchegoin, P. G. Surface enhanced Raman scattering enhancement factors: a comprehensive study. *The Journal of Physical Chemistry C* **111**, 13794-13803 (2007).

3 Fu, W. L., Zhen, S. J. & Huang, C. Z. One-pot green synthesis of graphene oxide/gold nanocomposites as SERS substrates for malachite green detection. *Analyst* **138**, 3075-3081 (2013).
